# A Systematic Review of Recommender Systems and Their Applications in Cybersecurity

**DOI:** 10.3390/s21155248

**Published:** 2021-08-03

**Authors:** Aleksandra Pawlicka, Marek Pawlicki, Rafał Kozik, Ryszard S. Choraś

**Affiliations:** 1 ITTI Sp. z o.o., Rubież 46, 61-612 Poznań, Poland; mpawlicki@itti.com.pl (M.P.); rkozik@itti.com.pl (R.K.); 2 Institute of Telecommunications and Computer Sciences, UTP University of Science and Technology, 85-796 Bydgoszcz, Poland; Ryszard.Choras@utp.edu.pl

**Keywords:** recommender systems, collaborative filtering, cybersecurity

## Abstract

This paper discusses the valuable role recommender systems may play in cybersecurity. First, a comprehensive presentation of recommender system types is presented, as well as their advantages and disadvantages, possible applications and security concerns. Then, the paper collects and presents the state of the art concerning the use of recommender systems in cybersecurity; both the existing solutions and future ideas are presented. The contribution of this paper is two-fold: to date, to the best of our knowledge, there has been no work collecting the applications of recommenders for cybersecurity. Moreover, this paper attempts to complete a comprehensive survey of recommender types, after noticing that other works usually mention two–three types at once and neglect the others.

## 1. Introduction

The digital revolution gave birth to cybercrime, and brought about the concerns about the security of data, privacy and other digital assets of citizens. Cybersecurity has become even more crucial at the outbreak of the Coronavirus (COVID-19) pandemic, when millions of people were forced to turn online almost overnight, without prior knowledge or experience. Never have so many people been so vulnerable to cyberattacks and online mischief [1].

In the cases when one must make choices without sufficient knowledge, or experience of alternatives, one used to rely on advice and recommendations from other people. It came in several forms, from face-to-face conversations to film and book reviews in magazines, and from letters of recommendation to printed book guides. It is a natural, social process that the so-called recommender systems try to augment and assist in, by giving personalized suggestions and preventing being overwhelmed with the amount of information [2].

**Figure 1:**
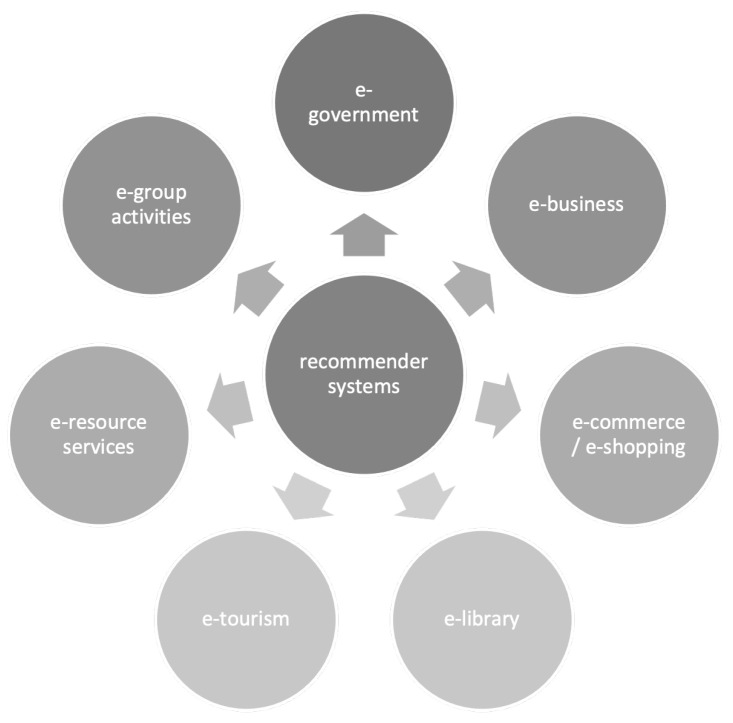
The application domains of recommender systems, based on the survey by [3].

Recommender systems have proven useful in innumerable applications, and each year, new ways of employing the techniques are proposed. In their survey on the development of recommender system applications, the authors of [3] grouped the possible implementations into eight “e-“ categories, e-government, e-business, e-commerce/e-shopping, e-library, e-tourism, e-resource services, and e-group activities. The application domains have been shown in Figure 1.

However, they do not mention the possible applications of recommender systems in cybersecurity.

In cybersecurity, the Security Operations Center (SOC) personnel faces vast amounts of data coming from a variety of sources, accumulated at rapid speeds. The constant flow of information often overwhelms the analysts, making timely and adequate response and mitigation unsustainable [4,5,6]. The so-called data triage automation is a well-known problem of SOCs [7], with the intensity of the domain and the characteristics of incident detection signals strongly degrading human performance [8]. However, the complexity of the task does not allow full automation at this point in time, which causes an ongoing discourse between the need for keeping human operators in the loop despite their physical limitations and the current state of possible automation [4,9,10].

On top of all those issues, cybersecurity is not only the SOCs concern—small businesses, especially in the domain of e-commerce [11] are prone to falling victim to cyberattacks, as they do not have the budget and the necessary skillset to protect their and their users’ assets [12,13].

One way of bridging the gap between the lack of resources allocated to cybersecurity, inadequate education, the limit of human capabilities and the technical state of automation in the domain is by building a system capable of recommending suitable cybersecurity response and mitigation measures. This solution would deload the human operator, and possibly make up for the deficiencies in education.

However, there have not been many cases of applying recommender systems in cybersecurity. Additionally, to the authors’ best knowledge, there has not been a survey gathering the cases where recommender systems were applied to aid cyberdefenders.

This is why a systematic review [14] has been conducted to identify the sources describing the applications or recommender systems in cybersecurity. The following work presents the results of the study.

The paper is structured as follows. In Section 2, the review process has been described in detail and the Research Questions have been raised. Following this, the concept of recommender systems is discussed in detail, and the authors gather the list of recommender types. It is then followed by an outlook on their drawbacks and advantages, usual applications, and concerns about their security. Then, against this background, the results of the literature review are presented, i.e., the existing implementations of recommenders in cybersecurity are surveyed, along with some proposals thereof, followed by the final conclusions.

## 2. The Conduct of the Study

The desire to gather and describe the applications of recommender systems in cybersecurity, has been the primary motivation for conducting the study. The fact the works on the applications of recommender systems tend not mention this possible use has also contributed to it.

**Figure 2:**
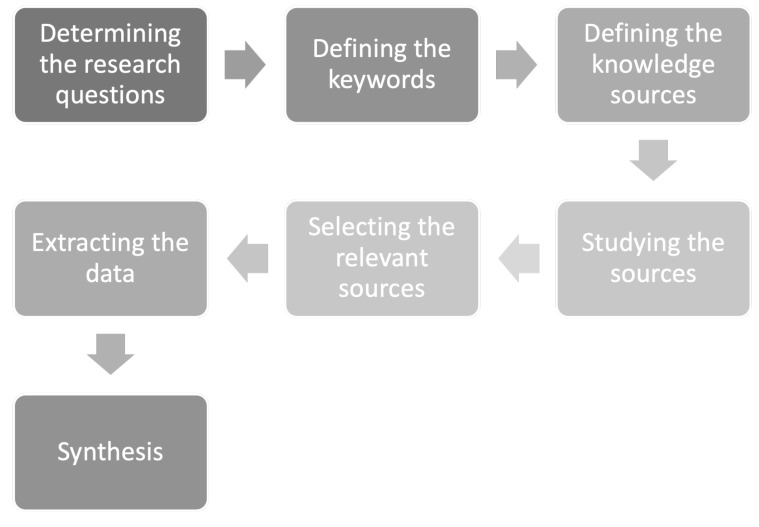
The pipeline of the study course.

The pipeline of the study course has been presented in Figure 2.

At the beginning, the following research question was defined.

RQ1: What is the current state of the art regarding the application of recommender systems for cybersecurity?

Additionally, during the study, it turned out that the analyzed works often did not agree about the system of dividing recommender system types. Conversely, some of them mentioned only the basic types (e.g., in [15,16]), or even fewer of them (like in [17]), while others mentioned other types in various combinations ([3,18,19], etc.). Thus, the authors of the study wished to aggregate as many types of recommender systems and another research question was formulated:

RQ 2: What is the actual, up-to-date and the most comprehensive division of the recommender system types?

The study contained in this paper gathers the applications of recommender systems in cybersecurity found in scientific literature. Before disclosing the cases, the context is set by completing a comprehensive survey of recommender systems, augmenting and unifying the taxonomies found across numerous previous surveys, also including the information found in method descriptions brought up in the investigated research pieces.

The study took place from February 2021 to May 2021. The literature items were gathered, analyzed and selected with a mix of bibliographic methodologies, including the snowballing method [20], pearl-growing [21] and citation searching and Preferred Reporting Items for Systematic Reviews and Meta-Analyses (PRISMA), all adjusted to fit the criteria of the PRISMA statement [22]. Pearl-Growing is a methodology of citation and subject search which involves finding a highly relevant piece of research and using it to find more relevant sources by extracting both the bibliography and relevant keywords, which can then in turn be used in another wave of searches. It is akin to the Snowballing method, which relies on consulting the bibliography of numerous research pieces to find other relevant papers, and then consulting the bibliographies of those to find even more relevant items. The weak point of those methodologies is that they focus on going backwards in time. To offset this circumstance, the authors employed Citation Searching. Citation Search uses the ‘Citation’ or ‘Cited in’ tab made available by the major publishers to find research papers which cite the investigated piece, a procedure which yields more recent items.

First, the keywords used for searching the items were determined. They were the combinations of the words “recommender systems” and “cybersecurity”, “threat intelligence”, or “attack mitigation”. Additionally, this set was expanded to include the alternative spellings: “recommendation systems” and “cyber security”.

Then, the publication databases were determined; the sources were thus searched in journal databases: Institute of Electrical and Electronics Engineers Xplore (IEEEXplore), SpringerLink, arXiv, Elsevier, Association for Computing Machinery (ACM) Digital Library and ScienceDirect. The more general sources such as Google Scholar were not taken into account as they mostly index the works from the aforementioned databases. The keywords were inserted in the search fields of the databases selected as sources. The initial, collective number of all the search engine hits was 1,700,067. The huge number of hits was the result of the fact that part of the search engines returned an immense number of false positives; as they were gradually less and less relevant, the browsing of the results stopped when the researchers decided the items became irrelevant to the study. The breakdown of the initial hits is presented in Table 1.

At this stage, the titles, keywords and abstract (if available) of the papers were analyzed.

The inclusion criteria for the papers were as follows: the papers had to describe the use of recommender systems in cybersecurity, be published in conferences or journals, and be written in English. During the study, several theses were found which appeared relevant, so they were also subjected to further analyses. The exclusion criteria were as follows: papers did not address the relation between recommender systems and cybersecurity, or were the duplicates of the previously identified items.

Altogether, as a result of the aforementioned bibliographic methods, 393 research items were marked as potentially relevant and further analyzed. Out of them, 86 works were selected for the survey due to their quality and appropriateness to this survey topics, among them 12 other surveys [3,15,16,17,18,23,24,25,26,27,28,29]. The papers most relevant to the application of recommender systems for attack mitigation are discussed in the Section 4.

The following section presents the concept of recommender systems and their types; this will serve as the background for the analysis’ results which will be introduced in Section 4.

## 3. What Are Recommender Systems?

The simplest definition of recommender systems is that they are programs attempting to recommend the best items to particular users, with the user’s interest in the item being predicted using the data on the items, the users, and the relations between them. Another definition emphasizes the fact that recommender systems assist and augment the social process of using recommendations of others, to make choices, when an individual lacks sufficient personal knowledge or experience of the possible alternatives [2].

The recommended items are usually particular products or services, while the users may be both individuals and businesses [3]. Recommender systems are built to effectively analyze the data and find out only the most relevant information from enormous volumes of data, thus avoiding the information overload and making the service as personalized as possible. An effective recommender system can “guess”, or “predict” the particular user’s interest or preference, based on the analysis of their behavior, or the behaviors of other users. This kind of systems have become an independent research topic in the mid-1990s [3].

The application of recommendation systems offers several significant advantages. In the online shopping environment, they enable easier finding of items, thus cutting the transaction costs, and ultimately, as they contribute to selling more products, they help yield more revenue [15]. However, although recommender systems are mostly known for the e-commerce applications, they have been found useful in multiple other domains. For example, in scientific online catalogues, they may enhance the experience, by recommending publications which are beyond the searches. The recommender systems which have entered collective consciousness, i.e., the ones that people are most often aware of are, e.g., the systems used by Netflix [30], YouTube [31], Amazon [32], or Hulu [33]. In general, recommendation systems have been widely accepted tools for boosting the decision-making processes in various domains [15]. The following benefits of the application of recommender systems have been listed by [23]: increased revenue, boosted client satisfaction, better-fit personalization, fulfilling the people’s need for discovery, and scrupulous reporting.

### 3.1. Basic Terms

Although there exist plenty of algorithms and techniques which fall under the umbrella of recommender systems, they all have several elements in common. According to [34], each recommender system contains the following three elements:

- items
- users
- transactions.

By items one understands the entity that is recommended by means of the system, i.e., its output [35]. Items have the attributes users are interested in. They also possess other structures, used to rate/value them. Then, the users are the ones, whose information is used by recommenders to make new item recommendations. Users have various goals and characteristics. Finally, a transaction means the potential interaction between a user and the system. The item rating is based on the set of transactions. In recommender systems, the information gathered from some transactions is used to make a new recommendation [34].

At the beginning of recommender systems, the features that researchers concentrated on were of two-dimensional, user x item nature. This was caused by the scarcer computational resources as well as by insufficient knowledge. Presently, additional data are gathered and used to make the predictions more accurate, such as demographic, temporal or social network data [36].

### 3.2. Creating a Recommender System: The Principles

When creating a recommender system, ref. [34] proposes for the process to take into account the following dimensions of the recommendation problem:

- “Users: who are the users of the system? What are their goals?
- Data: What are the characteristics of the data the recommendations are based on?
- Application: what is the application the recommender is part of? [34]”

### 3.3. Filtering Techniques

**Figure 3:**
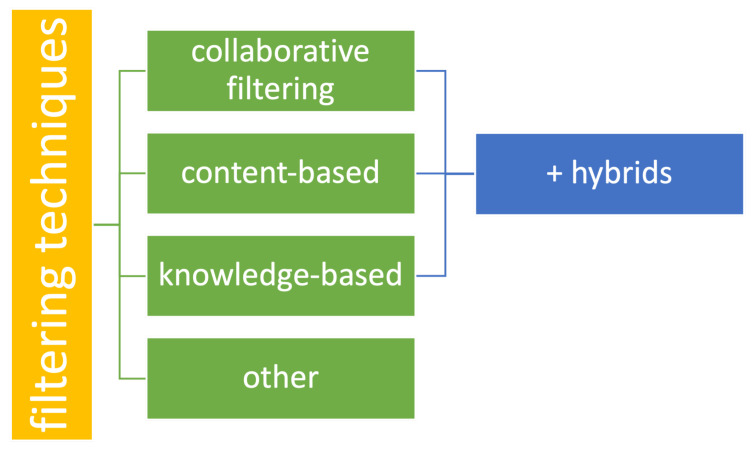
Recommender system types by filtering technique; the division devised by the authors for the sake of this work.

There are several techniques used for building recommendation systems. The most popular ones are: Collaborative Filtering (CF) [37], Content-Based (CB) [38] and Knowledge-Based (KB) [24]; they can also be combined to form various hybrids. The abovementioned types are the ones which most researchers agree on. However, there are other, less common methods that are mentioned by much fewer papers, or the researchers have different opinions on which group the methods should be classified into. This paper aims at mentioning at least most of the less-known and -used techniques, and lists them separately from the main three types. Figure 3 shows the division applied for the sake of this work.

#### 3.3.1. Collaborative Filtering (CF)

**Figure 4:**
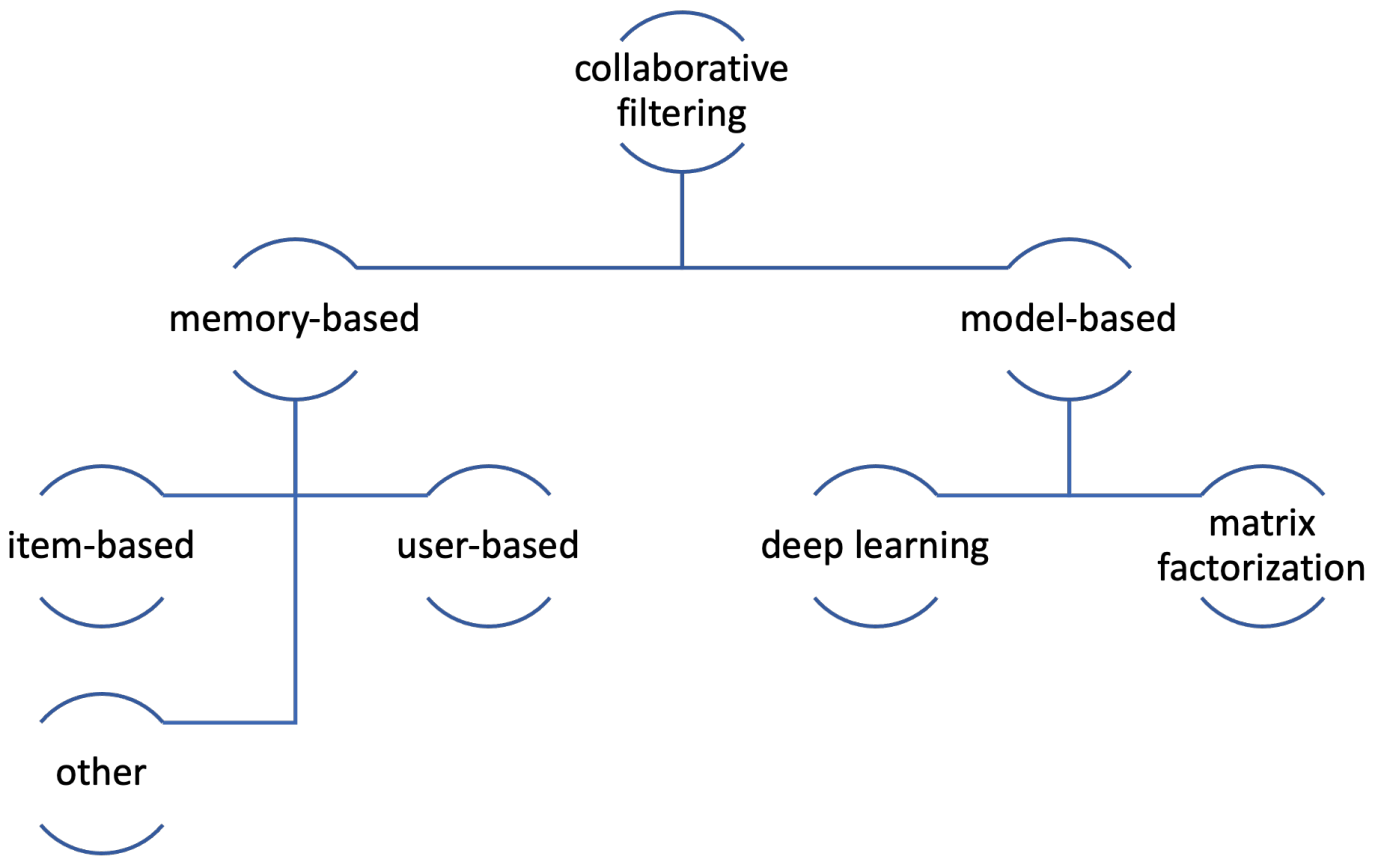
The kinds of collaborative filtering discussed in this work.

Collaborative filtering was called “the most mature and the most commonly implemented” technique [15]. It mostly been applied in the analysis of customer preferences and purchase patterns, and other content which is unable to be described by metadata, such as music or movies. The basic task of collaborative filtering is to find users with similar preferences or tastes based on their opinions, the so-called nearest neighbors; in other words, to search for the items that the user may like, based on the reactions of the users who have a similar taste. Simply put, collaborative filtering analyses big groups of people and aim at finding much smaller sets of users who share their preferences with the user of interest. The items which were liked by the people from the set are the basis for building a ranked list of recommendations; the similarity of users may be calculated in several ways and so may be the recommendations, based on this data [39]. Figure 4 presents the types of CF presented in this paper.

In collaborative filtering, the results may either be predictions or recommendations. The former come in the form of a numerical value *Rij*, meaning the prediction for the score of the item *j*, while *i* expresses a particular user. Recommendations of the latter type come as a list of N items which will most likely be selected by a user [15].

**Figure 5:**
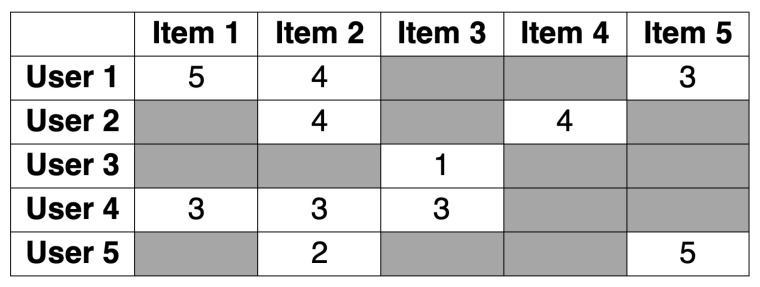
A sample user–item matrix.

As this kind of filtering is based on the actions of the existing users, i.e., the recommendation is produced depending on the observations of the actions a new user takes and comparing them with the actions and ratings of the existing users, in most CF techniques, the generated preferences are based on a user–item matrix [19]. Such a matrix is built of a set of items and a set of users who reacted to some items. As [39] explains, this reaction may be either explicit (numeric rating, or expressing “likes” or “dislikes”) or implicit (i.e., the user’s behavior: clicking the link, the fact of viewing the item, adding it to a wishlist, or spending a certain amount of time on the item). A sample of a matrix of this kind has been presented in Figure 5.

In this matrix, rows represent the ratings users gave, while columns mean the ratings that a particular item received. Therefore, the third user has rated the third item and gave it a rating of 1, etc. As it is hardly possible for every user to rate every possible item, and realistically, a person usually rates a few items, most matrix cells will remain empty. If most cells of the matrix are empty, it is called sparse; if most cells are filled, then it is said to be dense [39].

Based on the data, the nearest neighbors are uncovered. The collaborative recommender systems assume that those strongly correlated users will have an interest in similar items. Such users are grouped into the so-called neighborhoods. Consequently, this kind of systems will recommend buying the items which one of the similar users bought and the other did not, with the prediction value for that item being drawn from the proximity of this neighbor to the average rating of this user; in other words, a user is suggested to choose the items that other users in their neighborhood found favorable [15].

To calculate the similarity between users or items, several methods may be employed. One of the most widely used ones is the Pearson’s Correlation Coefficient (PCC); it has also been proven to perform better than other metrics by several researchers (e.g., [40]). It is used to determine the strength of the linear relationship between two variables. It is expressed by Equation (1) [36]:

(1) $$sim\left(u,v\right)=\frac{\Sigma i\epsilon I_{uv}\left(r_{ui}-{\bar{r}}_{u}\right)\left(r_{vi}-{\bar{r}}_{v}\right)}{(\sqrt{\Sigma i\epsilon I_{uv}{\left(r_{ui}-{\bar{r}}_{u}\right)}^{2}\Sigma i\epsilon I_{uv}{\left(r_{vi}-{\bar{r}}_{v}\right)}^{2}}}$$

where $I_{uv}$ is the set of the items that both users *u* and *v* have rated, while $r_{ui}$ and $r_{vi}$ are the ratings that both users gave to the item. In this metric, the similarity is measured on a scale from −1 to 1, where −1 is a perfect negative correlation, 0 means no correlation, while 1 represents a strong positive correlation. The users who display high positive correlation values have bought very similar items.

Another function which is commonly used in similarity calculations is the cosine similarity, shown in Equation (2) [36].

(2) $$sim\left(u,v\right)=\frac{\Sigma i\epsilon I_{uv}r_{ui}r_{vi}}{\sqrt{\Sigma i\epsilon I_{uv}r_{ui}^{2}\sqrt{\Sigma i\epsilon I_{uv}r_{vi}^{2}}}}$$

Cosine similarity calculates the similarity by measuring the cosine angle between two vectors of an inner product space [41]. The input parameter *u* is a user, while *v* is an item. If the item matches the user exactly, the angle between two vectors would be 0 degrees, the cosine of which would be 1. On the contrary, the angle between the most “dissimilar” vectors would be 90 degrees, leading to the score of −1. All the values between −1 and 1 represent intermediate (dis)similarity [35].

Other algorithms used for calculating similarity are, for example, the Euclidean distance, Manhattan distance, Spearman correlation, entropy-based uncertainty, men-square difference, Minkowski distance, etc. [35,41].

Collaborative filtering approaches fall into two main categories, memory-based and model-based recommendations. The memory-based techniques can be further divided into item-based and user-based ones; this paper also discusses additional, less-known ones. In turn, by the model-based techniques one usually means matrix factorization algorithms and deep learning [42].

##### Memory-Based Collaborative Filtering

In the memory-based collaborative filtering, the recommendations are made based on the whole collection of the rated items. List of recommended items is built based on the items chosen and rated frequently by the users belonging to the same group. These techniques are composed of the following steps: data pre-processing, selecting the set of K users/items that show the greatest similarity to the particular user/the items they have already rated (a.k.a., selecting the neighborhood) and computing the recommendations, i.e., generating predictions and listing the top-N of them [41]. The similarity between users or items can be measured using several metrics, usually the Pearson correlation coefficient and cosine similarity.

- User-based CF
  By this approach, the users are matched by the recommender engine based on their taste in the product in question [43]. Simply put, in the user-based collaborative filtering, the user U and the set of users similar to them are selected. Then, the rating for an item is searched for; the user has not rated the item. By choosing N of the similar users who did rate the item, the rating is then calculated.
- Item-based CF
  This type of recommendation is based on the concept that customers tend to choose items similar to the ones they expressed an interest in, and at the same time will not buy the items they are not interested in. In this kind of system, the user–item matrices are used as an input for finding the relations among various items. The analysis of how the items interact is the basis for generating a personalized recommendation.
  The item-based algorithms tend to perform better than the user-based ones, as the latter ones are known to have scalability issues, i.e., when the user–item matrix is of substantial size, the computational time becomes very considerable. As the relations between items are more stable than those among users, item-based algorithms usually need less computational time to make correct predictions, or the computations may be performed offline. Then, the rating may be calculated from the Pearson’s correlation coefficient and the N nearest neighbor.
- Other types of memory-based collaborative filtering
  Very seldom, the researchers classify other methods as memory-based collaborative filtering, such as Predictability Paths, cluster-based smoothing and trust inferences in [41], and so on.

Collaborative filtering faces several challenges, one of them being the so-called sparsity. It results from datasets lacking big amounts of data, which makes it hardly possible to make accurate recommendations. Another issue is called a cold start. It arises after a new user has rated too few items for the matrix to make accurate predictions based on the available ratings. It can also happen when the ratio of items to users is very high. In such a case, it may be impossible for the user to rate enough items for the recommender to make a prediction. Both sparsity and cold start result from the fact that it is hard to match users with a low number of ratings to similar neighbors. The possible solution to these problems is, e.g., to apply the spreading activation technique. It consists of turning the data from the matrix into a graph and finding the relations between users and items. In the graph in which distance is the number of edges between the item and the user, the recommendations are built depending on how close the item is from a user [44]. Another possible solution makes recommendations based on similar user ratings and probability. The accuracy of suggestions improves using the information which does exist for the users [45]. Finally, the accuracy may be improved by using a hybrid system, i.e., exploring other data sources, such as item attributes or demographic data [19].

Another issue which can directly impact the recommendation process are the so-called grey-sheep customers, i.e., the customers whose tastes are either unique or exotic. Thus, the recommendations made for them could be of poor quality, and, conversely, the grey-sheep customers negatively affect the recommendations made for all the other customers [46,47].

##### Model-Based Collaborative Filtering

Some of the drawbacks of the item-based and user-based models have been addressed by the model-based ones. The Netflix competition in 2006–2009 was said to spark interest in this type of technique [48]. The advantage of this approach lies in the fact that the data are first processed in an offline manner, subsequently leading to building a model. This makes it possible to avoid real-time computations. Additionally, by this approach, machine learning methods are used to find user ratings of unrated items. Thus, the model-based recommenders, after training, can make very accurate recommendations [25]. However, it must be noted that without sufficient training, the models may be less accurate than the memory-based methods [41]. Generally speaking, the model-based approach involves reducing or compressing a user–item matrix which is substantial in size, but sparse. The technique is called dimensionality reduction. It relates to the cases where just a small part of the available items has user ratings. Memory-based techniques are not able to generate accurate predictions in such cases; on the other hand, model-based algorithms are designed to find latent factors (i.e., implicit, product-specific features) that help predict the lacking ratings [49]. As the user–item matrix consists of two dimensions—the number of users and the number of items—the dimensions which are mostly empty contributes to boosted performance of the algorithm. The dimensionality reduction is usually performed by means of matrix factorization. It can also be done with autoencoders, clustering-based algorithms, etc.

- Matrix factorization
  It consists of breaking a large matrix down into a product of smaller ones [16,41]. The algorithms employed for factorizing matrices are Singular Value Decomposition (SVD), Principal Component Analysis (PCA), Non-negative Matrix Factorization (NMF), and so on [41]. Using the algorithms, the features may be extracted for every product that has been rated. Then, a comparison is made between them and the items which do not have any ratings and finally, based on this, the rating is predicted [35,36].
- Clustering-based algorithms
  Usually, by this type of algorithms, one means the k-Nearest Neighbors (kNN), a Machine Learning (ML) technique. The aim of this algorithm is to search for clusters of similar users, the similarity being based on the users’ past behavior (like their ratings, the items they had already bought, etc.). Although the user-based collaborative filtering is based on the same concept, with the kNN the similarities are found based on an unsupervised machine learning model. Additionally, the number of similar users is limited to k [42]. It is worth noting that some researchers argue this technique does not belong to the model-based recommenders, but rather, it should be classified as a memory-based one, as though it is a machine learning technique, it is of non-parametric nature [42].

#### 3.3.2. Content-Based Filtering

The content-recommendation systems are said to be most effective for recommending text-based items, such as documents, news items or web pages [15].

In the content-based recommendation systems, the predictions are made based on the data on the items and past actions of users, and not on the other users’ choices [15]. The concept behind this kind of filtering is that a user tends to buy future items which in some ways are similar to the ones they previously bought. The similarity of items is calculated based on several their features and/or attributes. The main challenge of this approach consists of the collection of the data about items. Lack thereof results in sparsity, as in the case of collaborative filtering. Although in content-based recommender systems there is no need for a vast number of users or item ratings (like in collaborative filtering), they require a proper amount of information to make accurate predictions. The features/attributes used in this type of filtering include the metadata or even the actual contents of documents [19]. Generally speaking, in CB recommender systems, two main techniques have been applied for making recommendations. One of them, the heuristic one, uses traditional tools such as cosine similarity measures. The other approach employs machine learning and statistical methods, using the past users’ data for learning the models which are then able to predict users’ interests [50]. With this method, when making a recommendation, a vector is built, in which 1 means a word is present within a document, and 0 indicates the document does not contain it. Following that, the vector is compared with other documents by the recommendation system. One of the challenges to this method is the fact that the vector is in favor of longer documents, and that the frequency of a word in the document is not taken into account [51]. To solve this problem, the analysis of documents should be made by means of the technique called Term Frequency-Inverse Document Frequency (TF-IDF). TF takes into consideration the number of appearances of a word within a document. In turn, IDF attributes greater weight to the words which are only present in one document, thus helping to emphasize the difference between it and other documents [52]. Other means of modeling the relations between the items could be probabilistic models, such as the Naïve Bayes classifier [53], decision trees [54] or neural networks [55]. In these techniques, the model is learned using the machine learning or statistical analysis techniques.

The recommendation process begins with gathering the information on and the ratings of the items previously bought by a user. Then, the system searches for similar items. The similarity is determined as follows: using a similarity calculation, the items are collected into neighborhoods; in other words, neighborhoods are built by making a comparison between new items and the items already in the inventory. If a user bought an item, it counts as a vote for the neighborhood associated with the item. An item ought to be recommended to a user if they rated highly k of the nearest neighbors. This method is said to require relatively little data to make accurate predictions, and to be easily adaptable [51], i.e., if there are changes to the user’s profile, the recommender can swiftly adjust the recommendations [15].

It is helpful to keep long-term profiles of users, as it makes the recommendations more accurate, although short-term profiles are useful as well, e.g., when users tend to grow new interests in items. Apart from the problems mentioned above, the content-based filtering may face other challenges. The type and quality of the data associated with the items is of vital importance to CB recommenders. If the item is not text-based, extracting its features may prove to be a daunting task. In turn, with the text-based items, the systems consider words only, while other, more subjective attributes are neglected. A possible solution could be entering the attributes in a manual way; however, this solution is not entirely realistic due to the fact how time- and resource-consuming it is. The CB recommendation systems may experience sparsity as well, when there are not enough item attributes. There is a concern to be had if the content-based system begins to make suggestions which are too similar. For example, it may recommend a user the same item they bought previously, but from a different company. To avoid this kind of problem, the system may either be supplied with some diversity, or the items which are too similar must be excluded, by means of a filter [56]. It is also possible that a content-based recommender system experiences the cold start issue; however, as this type of recommender system needs just a few ratings or pieces of information on past actions, the problem is not as severe as with the collaborative filtering recommenders [51]. Lastly, content-based filtering may sometimes suffer from overspecialization, i.e., the situation in which the system starts recommending items which are very similar to one another, without suggesting novel items [50].

#### 3.3.3. Knowledge-Based Filtering

As [57] put it, knowledge-based recommenders are those that use different knowledge sources from the ones used by collaborative filtering and content-based recommender systems. Unlike the recommendations described before, the knowledge-based ones do not rely on the user–item data. Their predictions are based on explicit rules about the problem domain, as well as on the attributes of items. They do not track the actions of users and do not collect ratings; instead, this type of system gathers specific requirements from the users. Therefore, there is no problem with sparsity, even in the case of the seldom-bought items [57].

Generally speaking, knowledge-based recommenders may be divided into two approaches, constraint-based and case-based ones. Some researchers, such as [58], classify utility-based as belonging to the knowledge-based recommenders. For the sake of this paper, utility-based recommendation systems have been discussed separately.

The constraint-based recommenders are called this way, as they in fact compare the attributes of an item within the constraints, i.e., the requirements that the users give, or the constraints from the product domain. In other words, in this method, the recommendation equals satisfying the constraints, with the products which fulfil the constraints being the good recommendation [58]. This type of recommendation may also be made using a conjunctive query over the product database. In this approach, the user requirements for attributes are connected and a conjunctive query is created. Following this, a database query is made, and the items meeting the constraints are returned.

There are also case-based recommendations systems. They make predictions based on the similarities between items and the requirements. According to [59], the distance similarity of an item is dependent on the sum of all the similarities of attributes weighed by the requirements. Consequently, the distance between an attribute and a requirement is what the other items of interest depend on. In other words, local similarity is used for finding similar items. It is found by dividing the distance of the attribute of the item from the desired attribute by the total range of the attribute. Other case-based recommendations are made using a query-based paradigm, which is effective as long as users have specific requirements. The abovementioned case-based recommenders in fact depend on suggesting items that are close to the requirements, and do not need to fulfil all of them.

It is important to mention that although, following [60], this work classifies case-based recommenders as the knowledge-based ones, there are researchers such as [61], who classify them as content-based recommenders instead, or think of them as being a bigger, more general group, encompassing content-based and knowledge-based recommenders [62].

The limitations of the knowledge-based recommenders result from the inability to meet all the requirements the user has given. This may result in giving a null response. To avoid this, the system should be designed to suggest the users to relax the requirements, or even do it automatically. This way, the system is eventually able to suggest an item which is as close as possible to fulfilling the original requirements. Another way of making this type of system to give better predictions is to use a divide-and-conquer algorithm QuickXPlain [63]. The algorithm identifies the conflict between the user requirements and the potential items, i.e., constraints that the system is not capable of fulfilling.

#### 3.3.4. The Comparison of the Three Main Filtering Approaches

Each of the three aforementioned methods has its own advantages and flaws. The main, most prominent advantages and disadvantages, as well as the applications the techniques are the most suitable for, have been shown in Table 2.

#### 3.3.5. Hybrid Recommender Systems

As [3] remarks, the three main types of filtering play a dominant role in the majority of applications today; however, as the issues characteristic of the three aforementioned filtering techniques influence the recommendation’s quality in a negative way, hybrid filtering has been proposed [67,68,69].

The so-called hybrid recommender systems are usually based on the three main types of recommender systems. They can make more accurate predictions, as they combine different approaches to gather information. The final results depend heavily both on the used algorithms and the method of hybridization, i.e., the way and order in which the outcomes of an algorithm relate to the other ones. The recommenders that suffer from sparsity, i.e., CF and CB, are better for solving the issues where there is an abundance of data. On the other hand, knowledge-based recommenders cannot find associations between users and items. CF and CB systems can adjust to the changing needs of users, but the former systems outperform the latter ones in the cases when there is a lack of item attributes. Conversely, content-based ones can work better even without having a great number of user–item ratings to analyze.

To exploit the strengths of the systems, and not rely on their weaker points, hybrid recommenders are constructed to use various techniques on the same dataset. Afterwards, the resulting data are combined to make the final recommendations. For the combinations to give valid results, they must be given static weights. The weights may be influenced and changed, e.g., to reflect the users’ feedback.

One such hybrid might be a system which bases the used technique of computing recommendations on a given context/situation. Such a system is called a switching hybrid. If there were sparsity, it would first use a knowledge-based system and then, after the users’ rating, it would switch to collaborative filtering, and so on. The decision if and when switch the technique may be made based on the fact if the default configuration is able to give a valid result, or not [19,70].

A comprehensive list of the various hybridization techniques has been presented in Table 3.

Besides the techniques mentioned in the table above, there are also other manners of combining the filtering methods. As [71] remarks, it is possible to, e.g., make a unified recommendation system, while treating user rating prediction as the issue of machine learning, with probabilistic latent semantic analysis, or combine the similarities in a unified kernel space, where the predictions are made based on support vector learning, etc.

Another approach to combining recommendation methods is by applying graphs. In a database of this kind of a recommender system, data are contained in nodes; their edges are linked together. The links representing the relations may be either weighted or unweighted. Thus, the relationships between nodes are easy to retrieve, especially if the entities in the system are strongly connected [72]. Although it may take slightly more time to compute [73], this method is intuitive and available, and helps overcome the issues such as data sparsity [71]. The graph-based recommendation systems have already been tested in various applications, such as in a digital library [74], collaborative ranking [75], and making recommendations of drugs [76], books [73], and movies [77], and so on. Most of the aforementioned solutions are based on the Neo4j graph data platform; it has also been deemed the best choice among graph databases by [78].

#### 3.3.6. Other Types of Recommender Systems

There are several other, more specialized and thus less widespread recommender system types. It must be noted that there is no common agreement as far as the ontology is concerned, i.e., some of the types mentioned below are classified as belonging to the abovementioned groups by some researchers. This may be due to the dynamically changing domain and state of the art, or the fact that several methods show features which may belong to more than one group/type. In addition, several filtering types have been distinguished according to the technology applied and not the features taken into account when making recommendations; thus, some overlapping occurs, or the same technique is classified as more than one category.

- Computational Intelligence-based Recommendation Techniques
  Sometimes called CIRS, the computational intelligence recommender systems are the ones which include Artificial Neural Networks (ANN), Bayesian techniques, clustering techniques, genetic algorithms, fuzzy set techniques, etc., in their recommendation models. Bayesian classifiers solve classification problems based on probabilistics. They often are part of model-based recommenders, or help create a model for the content-based recommenders. With a Bayesian network being used for recommendations, the nodes correspond to items, while the states correspond to all the vote values possible. Thus, each item in the network will have a set of parent items—they will be its best predictors [3].
  Artificial neural networks have also been used as part of recommendation engines. For example, ref. [79] have applied one in a personalized TV recommendation system. They trained an ANN of three layers with the back-propagation method. A hybrid movie recommender was presented by [80]. The trained ANN representing the preferences of individual users was responsible for content filtering.
  To make the computational cost of finding k-nearest neighbors lower, clustering may be applied. Clustering consists of assigning items to groups. This way, the items within groups are more similar than the ones in other groups. With recommender systems, this may result in, e.g., smoothing the unrated data for users, by predicting the unrated items from a group of related items. Additionally, with the assumption that the nearest neighbor is within the Top-N most similar clusters to the active user, there is only the need for selecting the nearest neighbors in the Top-N clusters. This results in greater scalability of the system [3,81]. Furthermore, the technique can help tackle the cold start issue, by grouping items [82].
  Genetic Algorithms (GA), i.e., stochastic search techniques, have mainly been applied in K-means clustering, for improved online shopping market segmentation, such as in [83]. Similarly, ref. [84] have used a GA method for obtaining optimal similarity function. Finally, several techniques based on the fuzzy set theory have been used to handle the non-stochastic uncertainty, e.g., the information being imprecise, or the classes of objects not being sharp enough [3].
- Social-network-based recommendations
  The rapid increase in the social networking tools has directly resulted in social network analysis becoming an important part of recommender systems. Recommender systems offer the possibility for the users to make social interactions among one another, such as comments, adding to friendlist, etc. Based on these interactions, recommendations can be made. The social network recommendations rely heavily on the concept of “trust”. In human interactions, a person’s decision (to buy something) is more likely to be influenced by friends’ opinions than by an advertisement. Trust, i.e., the level of how one user trusts others concerning a product, is helpful in making predictions where the data on similar neighbors would be too sparse otherwise. Indeed, a positive correlation between trust and user similarity has been found scientifically [85]. In addition, the authors of [3] discuss other social interactions and relations which are used for making recommendations, namely social bookmarks, physical context, social tag, “co-authorship” relations, “co-citations”, and more.
- Context-awareness-based recommendation methods
  In recommender systems, context is understood as any kind of information which may characterize a situation or an entity, such as a person, place or an object that is relevant to the user–item interaction [86]. Context may thus mean time or the company of other people. Applying context in recommendation process makes the results more personalized and appropriate. As [87] claim, the rating function is no longer two-dimensional, i.e., (R: User × Item → Rating); instead, it has become multi-dimensional (R: User × Item × Context → Rating).
- Group Recommender Systems (GRS; also called e-group activity recommendation systems)
  Group recommendations are a method of making group suggestions “when group members are unable to gather for face-to-face negotiation, or their preferences are not clears despite meeting each other [3]”. They are used for recommending films, music, websites, evens or travels. The process of clustering people into a group may follow several strategies, based on the research of decision-making or social choice theory, such as the theory of average, least misery, most pleasure, and so on [44], as well as the strategies of sum or approval voting.
- Demographic filtering
  Some researchers, such as [23,88], describe the demographic filtering as a separate filtering technique. By this method, the system gathers the information such as age, gender, education level, place of residence, as well as users’ opinions on items. Then, the similarities are found between the users’ ratings; finally, the data are filtered by users’ age or the area they live in. According to [18], these methods form similar correlations to the ones present in collaborative filtering, but unlike the collaborative and content-based techniques, they may not need a history of user ratings. However, they may raise some security issues, due to the nature of data they gather [23].
- Utility-based recommender systems
  Lastly, there are utility-based recommenders. In them, the utility of an item for a user is calculated, with gathered the users’ interest level in that attribute. As with the knowledge-based recommenders, the utility-based systems are not based on building long-term generalizations concerning the users. Rather, the recommendation is made based on the assessed match between the set of available options and the users’ needs. Specifically, the utility-based recommenders calculate the utility of each object to a user and then make recommendations based on that. The weight of the attribute may also be calculated by the system, lowering the load on users. To do so, the total utility must be determined. It is the sum of all the item values, i.e., the weight multiplied by the similarity function. The system returns a list of items ranked according to their similarity level to the user requirements [59]. There are various approaches to what makes utility and how to compute it, but the general idea is that the utility function should be based on item ratings that the users offered to describe their preferences [37]. One of the main advantages of this filtering technique is that the utility computation can be influenced by some non-product attributes (e.g., product availability). This way, for a user who needs to receive an item as soon as possible, such a system could enable trading off price against delivery schedule [18]. As mentioned before, utility-based recommender systems are either seen as separate method of filtering [18], or as being part of knowledge-based recommenders [19].

## 4. The Result of the Study—The State of the Art of Recommender Systems for Cybersecurity

This chapter aims at presenting the results of the literature study, by gathering the instances when recommender systems were employed in cybersecurity. This will allow formulating the answer to the Research Question 1.

In their work, Polatidis et al. remark that recommender systems had not been used for attack prediction before [89]. Thus, they have proposed a method which combines collaborative filtering recommendation systems with the methods of discovering attacks paths. The attack graphs were built based on data sourced from maritime supply chain infrastructure. Their tool was validated and evaluated experimentally, proving its effectiveness. It uses a “parameterized version of multi-level collaborative filtering method (…) although other methods could be applied according to the scenario and the available data [89]”. Their method first uses CF, and then the k-nearest neighbors are rearranged, by the similarity value and the number of co-rated items. More specifically, the tool first finds all the paths that could be used to perform an attack. Then, a recommender system is applied to predict what attacks might take place within this network. The method the authors used was a parametrized version of multi-level collaborative filtering, although they make a remark that other algorithms could be applied as well. By this method, after CF has been applied, the k-nearest neighbors list is rearranged, to reflect the value of similarity and how many items had been co-rated. For attack classification, the characteristics from the abovementioned method was used. The authors first employed classical CF using the Pearson Correlation Coefficient, as shown in Equation (3) [89].

(3) $$Sim\frac{PCC}{a,b}=\frac{\Sigma p\epsilon P\left(ra,p-\bar{r}a\right)\left(rb,p-\bar{r}b\right)}{\sqrt{\Sigma p\epsilon P{\left(ra,p-\bar{r}a\right)}^{2}}\sqrt{\Sigma p\epsilon P{\left(rb,p-\bar{r}b\right)}^{2}}}$$

There, *Sim* (*a*, *b*) relates to the similarity of users *a* and *b*, $r_{a,p}$ is the rating of user a for product *p*, $r_{b,p}$ is the rating of user b for product *p* and $r_{a,r}$ represent user’s average ratings, while *P* stands for the set of all products [89]. Then, the authors analyzed the similarity values and the co-rated vulnerabilities. Based on this, attacks were classified, on a scale from very high to very low. The last step consisted of checking whether there were any attack paths between the assets. The authors mentioned that they did not just use classical CF without additional parameters, as it would be less effective than the method they had proposed.

Lyons [19] also proposes using a recommender system in cyberdefence domain. The main goal of this effort was to make the Observe => Orient => Decide => Act (OODA) loop of cybersecurity defenders faster, chiefly by speeding up the decision part. They propose implementing existing, effective Intrusion Detection System (IDS) solutions with a recommender system to provide the best steps to take in a given situation, so that the IDS provides the information to the recommender system to predict the likelihood of certain events for given nodes of the network. The system returns a list of actions—the actions are ranked and the highest one is supposed to mitigate the most events that the system predicted are likely to happen. The input is provided by the IDS—Network Modeler proposed in [90]. It consists of three main parts: sensors, database, and modeler algorithm. The sensors are mostly Commercial-Off-The-Shelf (COTS) software besides the custom-written host monitoring (java-based program sending updates to the sensor machine). For observing the network, Snort and Nmap were used. The database is a combination of the various data gathered by the system, i.e., Snort, Nmap, vulnerability scores, host monitoring software, etc. The host monitoring software sends updates to the base on the sensor machine; the data such as CPU and memory usage, etc., it also controls the antivirus software for every host. The task of the modeler is collecting and classifying the data about the health of the network, i.e., determining if there is any threat to the network, by analyzing the data from all the sensors, as well as generating a list of possible actions which may be taken on client machines/firewall machines to mitigate the threat. (e.g., update operating system, upgrade application, create/delete user account, disable port, block source IP address, etc.). Then, the output XML file describing the network state is created so that every host receives data about the attacks, infections, user accounts, etc., as well as the actions which could be undertaken for that specific host.

The recommender system used in this model is of collaborative type; it uses the user-based nearest neighbor recommendation algorithm; the users and products being replaced with nodes and their attributes. The similarity between the nodes and the attributes for each node is calculated using the Pearson correlation coefficient. The values range from −1 = strong negative correlation to 1 = strong correlation. Then, based on the similarity values, the prediction is calculated. If the nodes are similar (i.e., have similar vulnerabilities, thus one may suppose they are prone to the same kinds of attacks), then the events occurring on them will result in higher predictions. As the author explains, “the predicted rating for nodes are the values used to determine vulnerabilities that the knowledge-based recommender system needs to consider when generating defensive actions”. Then, a knowledge-based recommender system is applied for making recommendations of defensive actions, with the selected paradigm being called a constraint-based recommender (The system depends on the previously set knowledge of the actions which are used to mitigate/counter certain cyberattacks). At the start of the recommender system, all the actions it knows are loaded from the XML file; the file is loaded only once, making the first computation time greater than the other ones. Various exploits may be mitigated by means of one action; this is why the proposed action ought to be able to counter the greatest possible number of the predicted attacks. Thus, the presented list of suggested actions is sorted according to the number of the mitigated attacks. However, in this solution, the final choice of the action is left to the cyberdefender. In this recommender system, all the possible actions which may be taken to mitigate the threat are considered.

After calculating all the action values, the system sorts them and presents as recommendations, in descending order. The author remarks that to find out which recommendation algorithm proves the most effective, it is necessary to observe multiple kinds of them. The so-called hybrid recommender systems comprise of the collaborative, content-based and knowledge-based; they may be combined using various techniques. The author believes that the nature of cyber threats requires one to use a knowledge-based recommender, as the collaborative and content-based ones will be affected by the issue of sparsity. Thus, in their system, the author has combined a knowledge-based recommender with a collaborative technique.

In their paper, Sula et al. [35] have proposed applying a recommender system to support the mitigation of the Distributed Denial of Service (DDoS) attacks, by helping in the decision-making process of the defender and suggesting the most appropriate cybersecurity solution. The system, called ProtecDdoS, takes into account the requirements of a customer, i.e., the service type (proactive/reactive), the type of a DDoS attack, the coverage region (city, country or even continent), deployment time (minutes, hours, days, weeks), leasing period, and the client’s budget. The three last parameters may also be given priority, e.g., if budget’s priority has been set to high, it will show the cheapest solutions first, while the remaining characteristics will be close to the selected ones. The recommendation engine uses the following similarity algorithms: cosine similarity, Euclidean distance, Manhattan distance, Minkowski distance and the Pearson correlation.

The client-side of the solution has an Application Programming Interface (API) implemented using React. All the abovementioned parameters can be set there. The attack types may be entered from the drop-down field, but can also be imported from attack log files. Once the user’s profile has been set, the recommendation is given, i.e., a list of suitable services is presented, along with their description. The recommendations are supported with visual representations of the results, in the form of plots, to provide clients with better grasp of how the recommender works. Moreover, to provide the maximum security, it is possible to check the service’s hash, to see whether it was manipulated or not and can be trusted. The users can add more services to the database, too.

The server-side of the solution was implemented using Flask 1.0.2 The recommendation process bases on two components: Service Helper and Recommendation Engine. The helper component is responsible for filtering irrelevant data from the dataset, according to the user’s preferred features. It also calculates the index of characteristics, by assigning an integer value to each characteristic taking variables into consideration. Then, the Recommendation Engine calculates the customer index and service index. Then, similarity score is calculated using various algorithms and a resulting list of services is returned, sorted by the similarity index.

Soldo et al. [91] have proposed a method of predicting future attacks/malicious activity from past behaviors, calling it the “predictive blacklisting”; blacklists being understood as logs of past attacks, attack sources, etc. The recommendation system part was inspired by the one used by Netflix. As the authors said, they “framed the problem as an implicit recommendation system, which paves the way to the application of powerful machine learning methods. (…). Given a set of attackers and a set of victims, a number *r* is associated with every (attack source, victim destination, time) triplet according to the logs: *r* can indicate, for example, the number of times an attacker has been reported to attack a victim over a time period. More generally, we interpret *r* as the rating, or preference, assigned by an attacker to a victim [91]”.

The architecture of their model consists of three algorithms blended in a linear way; a time series model, responsible for accounting for the temporal dynamics, followed by two neighborhood-based models. The first of the two latter models is a modified kNN model, it predicts the attack while concentrating on finding similarities between the victims who were attacked by the same sources, ideally at the same time. The other algorithm belongs to the co-clustering type; its goal is to discover a group of actors that attack a group of victims at the same time, and it does it automatically [91]. The method differs from a traditional recommender system in several aspects. First, the authors addressed the issue of the rating matrix varying over time due to the changes in attack intensity. In traditional recommender systems, the matrix remains static. Another significant difference is that in recommender systems, the users provide the ratings themselves. In the case of this tool, as the rating is made based on the attacks reported in the logs, it is implicit.

The tools were tested on real-life data from the dshield.org website, which captures hundreds of millions security logs from a great number of websites. The authors claim that their solution, when compared to the state-of-the-art methods, proves significantly more accurate and more robust against poisoning the dataset.

In their paper, Franco et al. [92] (as well as [40]) have presented the MENTOR system, which they describe as a “support tool for cybersecurity”. It was designed to be able to recommend the most suitable protection measures (such as specific anti-malware tools, firewalls, etc.), as the market for such services is booming and end-users may not be able to find the best solution themselves, especially when under cyberattack, needing an ad-hock response and thus in a hurry. The system can recommend both the most suitable prevention and mitigation measures and does it according to various demands, i.e., taking into account the profile of a customer, their budget, as well as the properties of an attack. The architecture is structured as follows: first, the Service Requestor gathers the data concerning the attacked infrastructure as well as the attack itself from the monitor logs. The relevant data are also stored in a database for future analysis. Then, the information is sent to the Extractor and the recommendation process begins. It comprises of several steps; first, the data are analyzed and the correlations with the attack type are found using the Classifier component, by comparing the data to previously identified and known attacks. This allows determining the best mitigation measure, by means of Service Aggregator, which contacts various service providers and gathers the information on available services and their features, such as the region they cover, their price, etc. Based on this information, a list of protection services that could be used is created; then, the Aggregator is queried by Retriever. It can address the clients’ demands fully or partially, providing the most desirable price, performance or technological solutions. Then, the data from the Retriever is sent to the Recommendation Engine. At this step, based on the requirements derived from the Retriever, it uses several different algorithms to find the most suitable solution. Lastly, the customer input is used by the recommendation engine to find out which solution from the list is the best recommendation.

The system’s recommendation process was assessed by means of four commonly used measures of similarity, capable of quantifying the similarity of two items: (1) Euclidean distance, (2) Manhattan distance, (3) cosine similarity and (4) Pearson correlation. Measuring the similarity in a geometric way is possible, as the available protection services and customers’ requirements are mapped as vectors in space. In other words, the similarity may be evaluated when their magnitude and attributes are presented as directions in space.

The recommendation process starts when an integer array is created by indexing the parameters required by the client, and each service. Then, the properties of each service are indexed. Next, the profile of a customer is mapped as the Y vector, while the protection services as the X vector. This constitutes the input for the algorithms assessing similarity. Lastly, a similarity dictionary is built of the ratings. Service ID serves as a key here; owing to this, it can be used for the purposes of exporting or plotting the similarity.

MENTOR’s prototype consisted of a web-based user interface and the Recommendation Engine. A customer may set the requirements and prioritize their demands (high => low) using a dashboard. The prioritizing affects the final recommendation, by neglecting the remaining, less prioritized demands. The Recommendation Engine is also capable of running without the dashboards; in such a case the system acquires necessary input through the MENTOR’s API. Additionally, the choice of the recommendation algorithm may also be made by the end-user. To make the choice easier, MENTOR gives the user various kind of information on the classification results, e.g., in the form of graphs plots, comparison of the vectors representing the profile vs. the vectors of each service, etc. It may be a significant decision, as the results can vary greatly, depending on the methods used. This is so, as the features of a service are shown as a vector in space, some properties (especially the variables of high-magnitude, such as price) may drastically change the vector’s direction in space and influence the rating. This in turn may result in the services specified in the client’s profile, and thus favored in the recommendation process, may turn out to be worse than other possible choices. This is true for the algorithms based on distance (Euclidean, cosine, Manhattan), therefore the authors suggest that the Pearson correlation may be more accurate here, as it shows invariance to the elements’ magnitude. Another suggested way of overcoming the problem is to group the vectors of the services for every attribute and then consequently make a comparison between the service attributes and client-specific attributes in a 1-1 way. Thus, it is the average rating of the service’s attributes which constitutes the final rating. However, the authors notice that the attributes of the services which perform better than those specified by the customer will receive worse ratings, so their suggestion is to rearrange the input attributes to reflect the best conditions possible. This way the algorithm will provide the best option, not the one which is the closest to the request of an end-user.

Esposte et al. [66] have proposed using a recommender system which would collect cybersecurity alerts gathered from various external sources and recommend them to any person with network administrator profile. The main idea behind this was that a network administrator may be flooded with security alerts, but not all of them are relevant. Based on the administrator’s preferences and ratings, the alerts could be filtered using a recommender system. An interesting assumption is to make sure that some items will always be recommended, even if the user has not provided any requirements yet. The system is a hybrid one; first, the general ranking scores of items are calculated using the Bayesian Average, then, the collaborative filtering and content-based filtering are applied. The greater the ranking scores are, the better ranked the item is going to be. Then, the ranking is computed again, after adding weights for the elements, e.g., adding the greatest weight to the critical votes, etc. In cases when there are no rated items, the most recent cybersecurity alerts are shown. Otherwise, collaborative filtering and content-based approaches are applied. In the CF part, the similarity of users to their neighbors is calculated using the Jaccard similarity coefficient. The greater the coefficient, the greater the similarity. Then, the list of top-N recommendations for a user is computed. The other list is created using the content-based method, where the user’s interest in items is used to weight them, and the items the user is interested in have their tags added to the user’s interest. The higher the weight of the item, the more interest the user has in it. After performing collaborative filtering and the content-based recommendations, a mixed hybrid approach is then used to come up with the list of the top-N recommended items.

As the authors say, their “work advances the state of the art in cyber security by proposing a new model for gathering relevant information on cyber security alerts based on recommender system methods [66]”. The model was evaluated in an offline experiment and the results show it could be applied in the cybersecurity alert recommendation process. It is worth noting that the authors redefine the elements of a recommender system to suit the needs of cybersecurity administrators: the “item” in this case means a security alert, and its content elements are “item attributes”. The “user” here is the network administrator. By a “transaction” in this context one understands the potential interaction between “users” and “items”.

Casey et al. [93] discuss a full implementation of a recommendation-verification system in the context of defense against malware. They argue that it is possible to employ machine learning methods in learning the trace features of malware families. They present the requirements such a system would have to meet, and emphasize the significance of its interpretability. In an experimental way they prove the feasibility of the solution they propose.

In their paper, Du et al. [94] present the problem of People-Readable Threat Intelligence (PRTI), the amount of information in which may be overwhelming for cyberdefenders. They notice that making recommendations in this particular field is challenging, as the data tend to expire and become outdated very quickly, traditional knowledge graphs are not really suitable for this purpose owing to large amounts of noise in data, and the language of PRTI is highly condensed and specific. Thus, they proposed a knowledge graph created specifically for the PRTI recommendation equipped with a denoising entity extraction module. Then, they propose a framework which uses a Knowledge-aware Long Short-term Memory neural network (KLSTM) for providing external knowledge for PRTI recommendation, using information from the knowledge graph. They prove experimentally that their method of combining a Latent Drichlet Model (LDA; a three-level hierarchical Bayesian model) topic model with a KG-aware LSTM proves effective and more accurate than in the case without external knowledge.

Sayan et al. [95] have presented the design of the architecture of an Intelligent Cyber Security Assistant (ICSA). Such a tool would aid human cyberdefenders in their tasks. The proposed architecture would detect attacks using machine learning and recommend the defense solutions. It gathers the network traffic data using existing monitoring tools. Then, the data are analyzed using the anomaly-based intrusion detection approach. The results are then used for making recommendations. The Intelligent Recommender Assistant (IRA) is a module that leverages machine learning methods for making recommendations. The authors decided to use the knowledge-based technique; thus, the knowledge scope is assessed, and knowledge base must first be built. Then, they apply feature engineering to transform the raw data into so that it better suits the predictive model. The learned model is then tested on unseen data, making recommendations concerning the mitigation of real-time attacks. The authors claim that through iterative machine learning, their system can make accurate predictions and expect it to keep learning, adapting and improving, which will make it possible to let it make automated or semi-automated cyberdefense actions.

Panda et al. have proposed a recommender system the aim of which would be to find which machine learning model is the most suitable for identifying attack models. The recommender system’s components were Naïve Bayes, Decision Trees, Ensemble learning, AdaBoost [96].

Gadepally et al. argue that recommender systems show promise in cybersecurity applications, as they might significantly lower the time of responding to the threat [97]. In the cybersecurity domain, the analysts must process massive amounts of information, which may lead to overlooking crucial data. According to authors, a recommendation system would help filter and prioritize information, as well as weigh multiple factors. They think that a recommender could be used to track hundreds of websites in an automated way, to learn from past user behavior. This would be used to prepare recommendations for the IT security team. If vulnerability’s severity and possible impact were assessed, they could be then used to make a recommendation concerning patching the vulnerability, e.g., if the patching might be postponed or should be immediate. Recommendation systems might also be used for tracking network anomalies, to draw the IT security teams’ attention to the possible issues which could be solved in a proactive way. Finally, the authors remark that (as of 2016), future work would be needed to adapt the recommender technologies to meet the specific requirements of the cybersecurity domain.

## 5. Discussion of the Results

This work has first presented a detailed overview of the concept of recommender systems, their types and techniques, advantages and disadvantages, security concerns, as well as possible fields of application, pointing it out that the use of recommender systems as an aid to cyberdefenders in hardly ever mentioned. Then, against this background, the state of the art of the applications of recommender systems in cybersecurity was indicated, gathered from the systematic review of the subject literature. The results of the conducted study helped answer the Research Questions.

### 5.1. The Answers to the Research Questions

The answer to Research Question 1: The comprehensive, broad study of the subject literature allowed the authors to conclude that recommender systems have indeed been applied for the sake of cybersecurity. They were also able to identify several specific applications, which have been presented in the previous Section of this paper. Most of the authors of the analyzed papers claim they were the first ones or one of the first ones to apply this technology to cybersecurity, but believe it shows promise and in the long run could effectively assist the human operators in the loop. They believe that recommender systems show promise as a tool for cyberdefenders, as they “have the potential in mission scenarios to shift computational support from being reactive to being predictive [97]”. It has also been remarked that more exploration should be made into using recommenders in the cyber domain [19].

The answer to Research Question 2: While analyzing the sources, the authors noticed that the papers did not present a unified taxonomy of recommender system types. By gathering and organizing the types presented in the analyzed papers, this work proposes a new, up-to-date list of recommender system types. Thus, this work has presented the most comprehensive list of the kinds of filtering among all the analyzed papers.

### 5.2. Threats to Validity

For the sake of this work, a substantial number of papers have been looked upon and considered for further analysis. Due to the fact that three of the five selected knowledge sources’ search engines returned hundreds or thousands of false positives, the authors analyzed the hits until the results ceased to seem relevant. Furthermore, the study let the authors identify about a dozen works showcasing actual implementations of recommender systems for cybersecurity. The investigated works come from different approaches and are not prone to comparison. It emphasizes the need for further research in this emerging field, a similar survey ran in a few years could probably help answer the research questions more thoroughly.

## 6. Conclusions

This paper has presented the results of a broad, systematic study of the potential use of recommender systems in cybersecurity. Several hundred literature items were marked as potentially relevant and then carefully analyzed. Several papers presenting the implementations of recommenders in cybersecurity were found and described.

All in all, the study showed that recommender systems could indeed be applied to support the human cyberdefender in their decisions, and contribute to a safer, more secure cyberspace.

This emerging field still needs plenty of research and in-depth consideration. It might also be beneficial to further explore enhancing the systems, e.g., by the application of graphs, or combining the method with other cybersecurity tools.

Furthermore, in the course of the study, a list of all the mentioned recommender system types was created, making it the most up-to-date and comprehensive division, as of this day.

## Figures and Tables

**Table 1:** Search engine hit breakdown.

	IEEEXplore	SpringerLink	arXiv	ACM Digital Library	Science Direct	
recommender system + cybersecurity	20	296	11	549,218	1881	
recommender system + threat intelligence	15	1165	8	562,218	12,413	
recommender system + attack mitigation	3	235	10	552,639	19,935	
total	38	1696	29	1,664,075	34,229	1,700,067

**Table 2:** The comparison of collaborative filtering, content-based and knowledge-based recommender systems; compiled by the authors based on the subject literature.

	Useful in Recommending:	Advantages	Disadvantages
Collaborative filtering	- products on shopping sites, - movie and TV shows, - articles on news sites, etc.	- as it does not rely on machine analyzable content, it does need to “understand” the complex item, to recommend it [64] - can identify cross-genre niches, - adaptive, improves over time [18].	- have problems with sparseness, scalability, synonyms, cold start [16], - “new user problem”—the user needs to make a few ratings for the system to start working, - “new item problem”—an item must be rated several times for the system to work, - the “grey-sheep” problem; when a user belongs to more than one group, the recommendations become inaccurate [24], - prone to attacks and manipulation [65]
Content-based	- movies and movie stars, - books, - articles, - restaurants, - places to visit, etc.	- the ability to recommend new items even if there are no ratings provided by users, - they are able to adjust the recommendations quickly even if user’s references change, - recommendations can be given without using the users’ profiles = better for privacy, - are quite explainable.	- overspecialized recommendations (the system may not recommend items which are different but interesting to the user), - limited content analysis (they need well organized user profiles and plenty of descriptive data on items before making recommendations, otherwise two different items described by the same features will be undistinguishable), - “new user problem” [24], - sparsity of data, - does not consider the quality of items [66].
Knowledge-based	- products, - services.	- good for complex and highly customizable items [60], - no large datasets needed, - does not suffer from the cold start, new item and grey-sheep problems, - recommendations are reliable, as domain knowledge is noise-free [24], - recommendations may be made even for products which are not bought/experienced often enough for other methods to work.	- the construction of the database is complicated and requires “considerable domain knowledge, and expertise in knowledge representation” [24], - psychological factors limit the recommendation system (e.g., users tend to construct preferences while learning about alternatives) [58].

**Table 3:** The hybridization methods, based on [43].

Hybridization Method	Description
Weighted	The scores/votes of all the available recommendation techniques are combined together to produce a single recommendation.
Switching	The system uses a criterion dependent on the situation to switch between recommendation techniques.
Mixed	Recommendations from more than one technique are presented at the same time.
Feature combination	Features from different recommendation data sources are thrown together into a single recommendation algorithm.
Cascade	One recommender produces a recommendation which is then refined by another technique.
Feature augmentation	Output (a rating/classification) from one recommender is incorporated into the processing of the next recommender.
Meta-level	The model generated by one recommendation technique is used as the input to another.

## Data Availability

Not applicable.
